# Cerebral Cortex Apoptosis in Early Aged Hypertension: Effects of Epigallocatechin-3-Gallate

**DOI:** 10.3389/fnagi.2021.705304

**Published:** 2021-08-12

**Authors:** Min-Huang Hsieh, Zhen-Yang Cui, Ai-Lun Yang, Nguyen Thanh Nhu, Shih-Ying Ting, Shao-Hong Yu, Yu-Jung Cheng, Yi-Yuan Lin, Xu-Bo Wu, Shin-Da Lee

**Affiliations:** ^1^Division of Endocrinology and Metabolism, Department of Internal Medicine, Jen-Ai Hospital, Taichung, Taiwan; ^2^School of Rehabilitation Medicine, Weifang Medical University, Shandong, China; ^3^Institute of Sports Sciences, University of Taipei, Taipei, Taiwan; ^4^Faculty of Medicine, Can Tho University of Medicine and Pharmacy, Can Tho, Vietnam; ^5^Department of Physical Therapy, Graduate Institute of Rehabilitation Science, China Medical University, Taichung, Taiwan; ^6^Department of Internal Medicine, Jen-Ai Hospital, Taichung, Taiwan; ^7^College of Rehabilitation, Shandong University of Traditional Chinese Medicine, Shandong, China; ^8^Department of Exercise and Health Science, National Taipei University of Nursing and Health Sciences, Taipei, Taiwan; ^9^Department of Rehabilitation, Seventh People’s Hospital Affiliated to Shanghai University of Traditional Chinese Medicine, Shanghai, China; ^10^School of Rehabilitation Medicine, Shanghai University of Traditional Chinese Medicine, Shanghai, China; ^11^Department of Physical Therapy, Asia University, Taichung, Taiwan

**Keywords:** EGCG, hypertension, neural apoptosis, neural survival, caspase-independent apoptosis

## Abstract

This study aimed to investigate cerebral cortex apoptosis on the early aged hypertension and the effects of green tea flavonoid epigallocatechin-3-gallate (EGCG). Twenty-four rats were divided into three groups: a control Wistar-Kyoto group (WKY, *n* = 8), a spontaneously early aged hypertensive group (SHR, *n* = 8), and an early aged hypertension with EGCG treatment group (SHR-EGCG, *n* = 8; daily oral EGCG 200 mg/kg—94%, 12 weeks). At 48 weeks old, blood pressures (BPs) were evaluated and cerebral cortexes were isolated for TUNEL assay and Western blotting. Systolic, diastolic, and mean blood pressure levels in the SHR-EGCG were reduced compared to the SHR. The percentage of neural cell deaths, the levels of cytosolic Endonuclease G, cytosolic AIF (Caspase-independent apoptotic pathway), Fas, Fas Ligand, FADD, Caspase-8 (Fas-mediated apoptotic pathway), t-Bid, Bax/Bcl-2, Bak/Bcl-xL, cytosolic Cytochrome C, Apaf-1, Caspase-9 (Mitochondrial-mediated apoptotic pathway), and Caspase-3 (Fas-mediated and Mitochondria-mediated apoptotic pathways) were increased in the SHR relative to WKY and reduced in SHR-EGCG relative to SHR. In contrast, the levels of Bcl-2, Bcl-xL, p-Bad, 14-3-3, Bcl-2/Bax, Bcl-xL/Bak, and p-Bad/Bad (Bcl-2 family-related pro-survival pathway), as well as Sirt1, p-PI3K/PI3K and p-AKT/AKT (Sirt1/PI3K/AKT-related pro-survival pathway), were reduced in SHR relative WKY and enhanced in SHR-EGCG relative to SHR. In conclusion, green tea flavonoid epigallocatechin-3-gallate (EGCG) might prevent neural apoptotic pathways and activate neural survival pathways, providing therapeutic effects on early aged hypertension-induced neural apoptosis.

## Introduction

Hypertension is considered as the common cause of mortality (ESC/ESH, 2018). In hypertension, the brain suffers oxidative stress and inflammatory processes, which lead to neurodegeneration characterized by the progressive loss of neurons (Gąsecki et al., 2013; Hou et al., 2018). Neural apoptosis was reported in either hypertension or aged hypertension (Mignini et al., 2004; Poulet et al., 2006; Li et al., 2012, 2016; Gąsecki et al., 2013). Neural apoptosis in hippocampi is not found in the young (16-week-old) hypertensive brain, is further enhanced in the mature (32 week-old) hypertensive brain, and remarkably augmented in aged (64-week-old) hypertension (Li et al., 2016). Neural apoptosis is promoted by several major apoptotic pathways, including Endonuclease G (EndoG) and Apoptosis-inducing factor (AIF)-related Caspase-independent apoptotic pathway, Fas-mediated Caspase-dependent apoptotic pathway, and mitochondrial-mediated Caspase-dependent apoptotic pathway (Yakovlev and Faden, 2004). EndoG is not only the mitochondrial specific endonuclease but also the Caspase-independent factor, released into the cytosol in brain damage (Yakovlev and Faden, 2004). AIF is a mitochondrial oxidoreductase, also discharged to cytosol in response to death stimuli (Yu et al., 2010). During neural apoptosis, the cytosolic EndoG and AIF translocate into the nucleus, activating the neural Caspase-independent apoptotic manner through large-scale DNA loss and chromatin condensation (Bastianetto et al., 2011). To the best of our knowledge, the alterations of EndoG and AIF-related Caspase-independent apoptotic pathway in the early aged (48-week-old) hypertensive brain have not been investigated yet.

Fas-mediated Caspase-dependent apoptotic pathway in the brain is commenced by the binding of Fas ligand (FasL) and Fas receptor, which in turn recruits Fas-associated protein with death domain (FADD). Then, FADD activates Caspase-8, followed by Caspase-3 cleavage to execute the neural apoptosis (Choi and Benveniste, 2004). Caspase-8 also cleaves Bid to form truncated Bid (t-Bid) which translocates to mitochondria, playing as the intracellular apoptotic signal from the Fas-mediated Caspase-dependent apoptotic pathway to the mitochondrial Caspase-dependent apoptotic pathway (Akhtar et al., 2004). In the early aged hypertensive cerebral cortex, the alterations of the Fas-mediated apoptotic pathway have still not been explored.

The mitochondrial-mediated Caspase-dependent apoptotic pathway in the cerebral cortex is promoted by Bcl-2 pro-apoptotic factors in which t-Bid induces the oligomerization of Bax, Bak, and Bad. Those factors (t-Bid, Bax, Bak, and Bad) augment the release of Cytochrome *C* from mitochondria to the cytosol (Akhtar et al., 2004). Cytosolic Cytochrome *C* binds Apaf-1 to activate Caspase-9 and then Caspase-3, promoting the neural apoptotic process (Czabotar et al., 2014). On the contrary, in the Bcl-2 family-related pro-survival pathway, Bcl-2 family pro-survival factors (Bcl-2, Bcl-xL, and p-Bad) prevent the activities of Bax, Bak, and Bad, thereby reducing the levels of cytosolic Cytochrome *C* and preventing neural apoptosis (Akhtar et al., 2004; Czabotar et al., 2014). Bad is phosphorylated to form p-Bad, then binds with protein 14-3-3, activating pro-survival activity (Akhtar et al., 2004). Previous studies showed that Bcl-2 and Bcl-xL were decreased whereas Bax, cytochrome *C*, and caspase-3 were not increased in young about 4 weeks old but increased in mature hypertensive brain indicating that hypertension activated the mitochondrial-mediated Caspase-dependent apoptotic pathway and suppressed the Bcl-2 pro-survival pathway in the brain (Li et al., 2012, 2016).

Sirtuin 1 (Sirt1), a key factor of mitochondrial biogenesis, has been proven to provide neuroprotective effects by regulating several mechanisms, including the activation of neural survival pathways (Pfister et al., 2008). The PI3K/AKT related pro-survival pathway is the main pathway which protects the brain against neurodegeneration (Ersahin et al., 2015). Once triggered by many trophic factors, the phosphorylated PI3K (p-PI3K) activates the phosphorylation of AKT (p-AKT), promoting the downstream components to control the neural survival in the brain (Brunet et al., 2001; Ersahin et al., 2015). Besides, Sirt1 has been reported to activate p-AKT to enhance neural survival and reduce neural apoptosis in previous studies (Li and Wang, 2017; Teertam and Prakash Babu, 2021). Furthermore, p-AKT enhances the phosphorylation of Bad to form p-Bad, activating Bcl-2 family-related pro-survival members such as Bcl-2 and Bcl-xL to prevent neural apoptosis (Brunet et al., 2001). A study showed that hypertension reduced the phosphorylation of AKT, which, thereby inhibited the PI3K/AKT related pro-survival pathway in the brain of Stroke-Prone Spontaneously Hypertensive Rats (Yoshitomi et al., 2011).

Epigallocatechin-3-gallate (EGCG)- C_22_H_18_O_11_, the primary flavonoids extraction from green tea are characterized by anti-oxidant and anti-inflammation properties (Singh et al., 2011; Li et al., 2019). EGCG was reported to reduce blood pressure (BP) and improve memory functions in hypertensive individuals (Wang et al., 2012; Yi et al., 2016). Besides, studies have indicated that EGCG was able to attenuate neural apoptosis after stroke or traumatic brain injury (Itoh et al., 2013; Nan et al., 2018), but no previous research investigated the effects of EGCG on the hypertension-promoted cerebral cortex neural apoptosis.

The effects of EGCG on neural apoptotic and pro-survival pathways in the early aged hypertensive brain have remained unclear. We hypothesized that cerebral cortex apoptosis on the early aged hypertension is enhanced as well as that EGCG might prevent neural EndoG and AIF-related Caspase-independent apoptotic pathway, Fas-mediated Caspase-dependent apoptotic pathway, and mitochondrial-mediated Caspase-dependent apoptotic pathway, as well as enhance Bcl-2 family-related and Sirt1/PI3K/AKT related pro-survival pathways, in the cerebral cortex under early aged hypertension.

## Materials and Methods

### Animals

The male normotensive Wistar-Kyoto rats (WKY, *n* = 8) and male spontaneously hypertensive rats (*n* = 16) at 28-weeks old were purchased. All of the rats were housed at 22–24°C, 12 h light/dark cycle. They were freely accessed the standard chow (Lab Diet 5001; PMI Nutrition International, Brentwood, MO, United States), water *ad libitum*. The procedures were approved by the Institutional Animal Care and Use Committee of China Medical University, Taichung, Taiwan.

### Animal Experimental Protocol

At 36 weeks old, spontaneously hypertensive rats were randomly allocated into a spontaneously early aged hypertensive group (SHR; *n* = 8) and an early aged hypertension with EGCG treatment group (SHR-EGCG; *n* = 8). Rats in the SHR-EGCG group were treated daily by oral TEAVIGO (EGCG 94%; 200 mg/kg/day, once/day, between 7 and 17 am, 12 weeks), whereas the WKY and SHR groups did not receive any treatment. TEAVIGO, a highly purified green tea extract with 94% EGCG, was purchased from Healthy Origins, Pittsburgh, PA, United States.

### Blood Pressure Measurement

The BP-98A non-invasive BP system (a tail-cuff method, Softron, Tokyo, Japan) was used to evaluate systolic BP (SBP), diastolic BP (DBP), mean BP (MBP), and pulse pressure (PP) of rats from 32 weeks old to 48 weeks old, 4 weeks once and after EGCG treatment for at least 1 h. In each instance, BP measurements (SBP and DBP) were repeatedly recorded three times, then the average of them was calculated and regarded as SBP and DBP in each rat. MBP was calculated based on formula [MBP = DBP + 1/3 (SBP–DBP)]. Pulse pressure is defined as SBP–DBP. Values of SBP, DBP, MBP and PP in each group were expressed as means ± SEM from *n* = 8 of different rats in WKY, SHR or SHR-EGCG group.

### Brain Tissue Preparation

Rats were weighed before they were anesthetized by inhaled isoflurane 2% in a 70% oxygen mixture. Brains were removed, washed in ice-cold saline, and weighed. The cerebral cortex was dissected and divided into two sections for TUNEL assay-DAPI staining and Western blotting.

### TUNEL Assay and DAPI Staining

A neutral formalin solution was used to fix the cerebral cortex tissues. The samples were deparaffinized and rehydrated, incubated with proteinase K, then washed three times with PBS, soaked in permeabilization solution and blocking buffer, followed by 02 washes with PBS. Then, the slides were immersed in terminal deoxynucleotidyl transferase (TdT) and fluorescein isothiocyanate-dUTP conjugated using the *in situ* Cell Death Detection Kit, Fluorescein (Roche Applied Science), followed by washing with PBS. The slides were mounted by using DAPI fluoromount G (Southern Biotech, Birmingham, AL, United States). TUNEL-positive nuclei fluoresced bright green at 450–500 nm. The DAPI-positive nuclei were fluoresced by blue light at 340–380 nm. All counts were independently conducted by two people in a blinded manner.

### Western Blotting Analysis

The cerebral cortex tissues were homogenized in tissue protein extraction reagent (T-PER, Thermo Scientific) with protease inhibitors cocktail (Roche Applied Science, Germany). The protein extracts were obtained and then centrifuged at 12,000 *g* for 40 min to collect the supernatant. The Bio-Rad Protein assay (Hercules, CA, United States) was used to measure the protein concentrations of the cerebral cortex tissue. Protein samples (100 μg/well) were cataphoresized on a 12% SDS-PAGE at 80 V in the first 20 min, 100 V in the next 2 h, followed by transfer to polyvinylidene difluoride membranes (Millipore, Bedford, MA, United States) at 100 V in 90 min. Membranes were blocked in 5% skimmed milk at 25°C for 1 h, then were incubated overnight at 4°C with primary antibodies which were diluted 1,000 times, including EndoG, AIF, FasL, Fas, FADD, t-Bid, Bax, Bak, Bad, Bcl-2, Bcl-xL, p-Bad, 14-3-3, Cytochrome C, Apaf-1, Caspase-8, Caspase-9, Caspase-3, PI3K, p-PI3K, AKT, p-AKT, α-tubulin (Santa Cruz Biotech), Sirt1 (Cell Signaling Tech, Danvers, MA, United States), and COX IV (Taiclone Biotech Corp., Taipei, Taiwan). We washed the immunoblots four times with TBST. The immunoblots then were immersed 1 h in the HRP-conjugated secondary antibody solution (Santa Cruz), which was diluted 5,000 times, followed by 04 washes with TBST. The protein intensities were visualized using an enhanced chemiluminescence HRP substrate kit (Millipore Corporation) and the Fujifilm LAS-3000 system (Tokyo, Japan). The density of the bands was quantified by densitometry using Gel-Pro Analyzer densitometry software (Media Cybernetics, Silver Spring, MD, United States). The protein levels based on density of the bands was compared among groups expressed as fold changes relative to the control group and mean values ± SEM (*n* = 6 of different rats in each group).

### Statistical Analysis

The data of bodyweight, brain weight, TUNEL-positive cell percentages, and protein levels among WKY, SHR, and SHR-EGCG groups were compared by one-way ANOVA with the *post hoc* test. To compare BP, we repeated the measurement of ANOVA which was used with two factors (group and week-old) and the Bonferroni-adjusted multiple comparison test. We used SPSS 22.0 software for analysis. *P* < 0.05 was considered as a significant difference.

## Results

### Experimental Animal Characteristics

**FIGURE 1:**
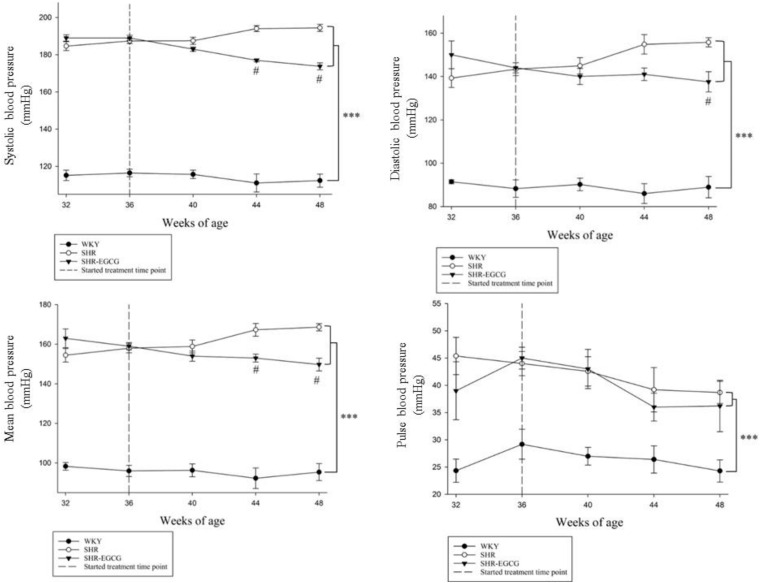
The line chart of systolic blood pressure (SBP), diastolic blood pressure (DBP), mean blood pressure (MBP), and pulse pressure (PP) levels with measurement time points in a control Wistar Kyoto (WKY) group, a spontaneously early aged hypertensive (SHR) group and an early aged hypertension with Epigallocatechin-3-gallate (EGCG) treatment (SHR-EGCG) group. Data were expressed as means ± SEM. ^∗∗∗^*p* < 0.001 in the comparison between the SHR group or SHR-EGCG group with the WKY rat group. #*p* < 0.05 in the comparison between the SHR-EGCG group and the SHR group.

Body weight and brain weight were unchanged among the WKY, SHR, and SHR-EGCG groups (Table 1). The heart rate in the SHR group was higher than those in the WKY group, whereas the heart rate in the SHR-EGCG group was lower than those in the SHR group (Table 1). During the entire study period, the levels of SBP, DBP, MBP, and PP in the WKY group were significantly lower than those in SHR and SHR-EGCG groups (Figure 1). However, after 8 weeks of EGCG treatment, the levels of SBP and MBP in the SHR-EGCG group were significantly reduced when compared to the SHR group (Figure 1). After 12 weeks of EGCG treatment, the levels of SBP, DBP, and MBP in the SHR-EGCG group were significantly reduced when compared to the SHR group (Table 1 and Figure 1). The PP levels were unchanged between the SHR and SHR-EGCG groups after 12 weeks of EGCG treatment (Table 1 and Figure 1).

**TABLE 1:** Characteristics of the experimental animal groups.

	**WKY**	**SHR**	**SHR-EGCG**
Number of animals	8	8	8
Body weight, g	403 ± 4	404 ± 6	412 ± 6
Brain weight, g	2.25 ± 0.02	2.29 ± 0.04	2.33 ± 0.04
Heart rate, beats/min	277 ± 10	387 ± 16*	341 ± 7*^#^
Systolic blood pressure, mmHg	112 ± 4	194 ± 2*	174 ± 2*^#^
Diastolic blood pressure, mmHg	89 ± 5	156 ± 2*	138 ± 5*^#^
Mean blood pressure, mmHg	95 ± 4	169 ± 2*	150 ± 3*^#^
Pulse pressure, mmHg	24 ± 2	39 ± 2*	36 ± 5*

*Values are expressed as means ± SEM.*

WKY, Wistar Kyoto rats; SHR, spontaneously early aged hypertensive rats; SHR-EGCG, spontaneously early aged hypertensive rats with Epigallocatechin-3-gallate (EGCG) treatment.

*p < 0.05 in comparison between the SHR group or SHR-EGCG group and WKY group.

*#*p* < 0.05 in comparison between the SHR-EGCG group and SHR group.*

### Neural Cell Deaths Comparison Among Three Groups

**FIGURE 2:**
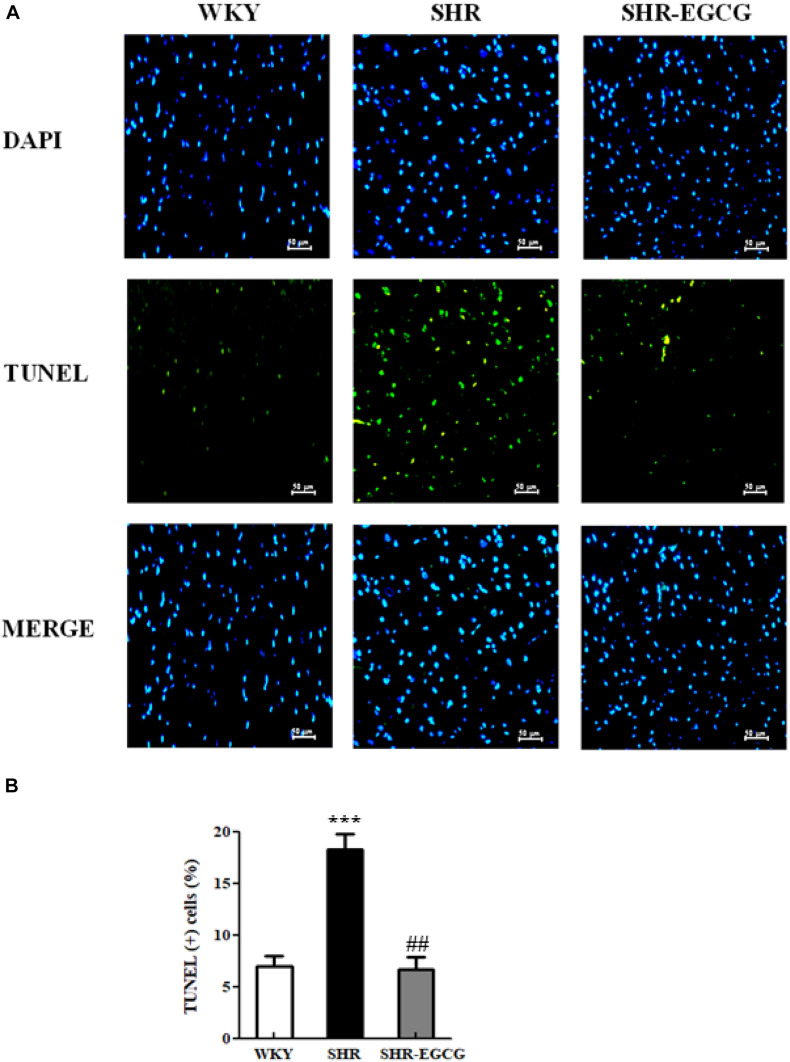
The TUNEL-positive neural cells in a control Wistar Kyoto (WKY) group, a spontaneously early aged hypertensive (SHR) group, and an early aged hypertension with Epigallocatechin-3-gallate (EGCG) treatment (SHR-EGCG) group. **(A)** Representative photomicrographs of DAPI staining (top, blue spots) and TUNEL assay (bottom, green spots) in cerebral cortex sections from the WKY, SHR, and SHR-EGCG groups (400× magnification). **(B)** The bar chart represents the percentage of TUNEL-positive neural cells relative to total DAPI-stained cells and expresses as mean values ± SEM (*n* = 6 in each group). ***p < 0.001 in the comparison between the SHR group and WKY rat group; ##*p* < 0.01 in the comparison between SHR-EGCG group and SHR group.

To determine the effects of EGCG on neural cell deaths in early aged hypertension, TUNEL assay and DAPI staining were carried out on cerebral cortex tissues from WKY, SHR, and SHR-EGCG groups. The number of TUNEL-positive neural cells in the SHR group were significantly increased when compared to the WKY group. On the contrary, the number of TUNEL-positive neural cells in the SHR-EGCG group were significantly reduced in comparison with the SHR group (Figure 2).

### EndoG and AIF-Related Caspase-Independent Apoptotic Pathway

**FIGURE 3:**
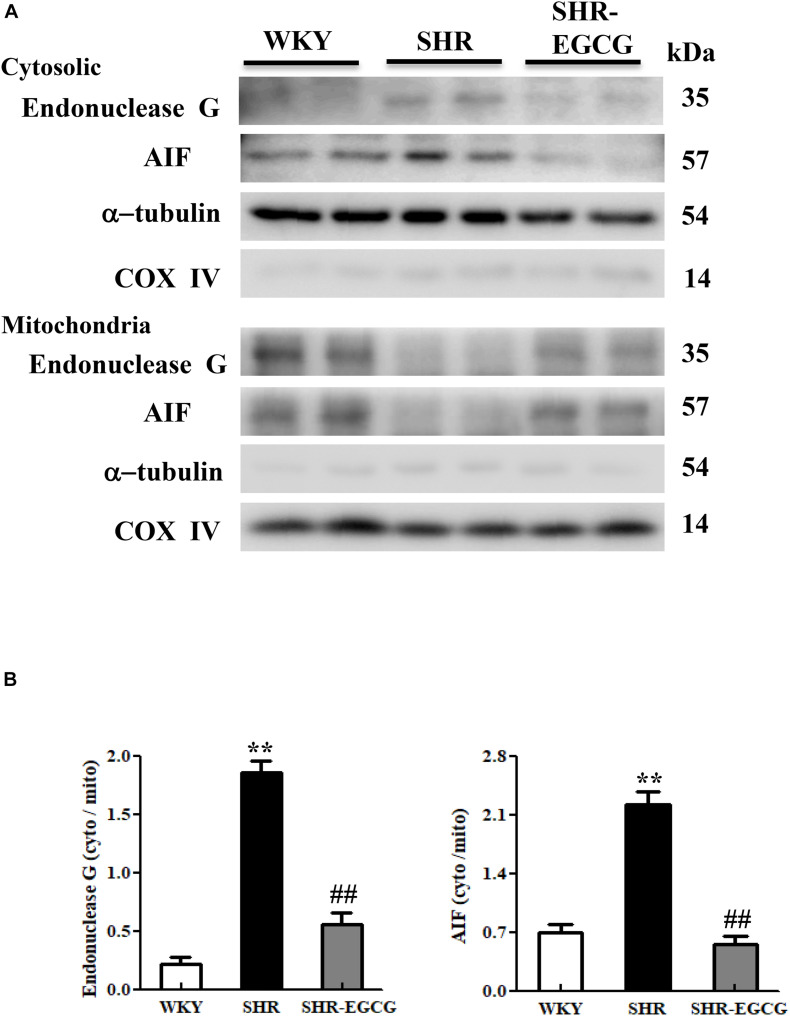
The Caspase-independent Endonuclease G (EndoG) and Apoptosis-inducing factor (AIF) apoptotic pathway in a control Wistar Kyoto (WKY) group, a spontaneously early aged hypertensive (SHR) group and an early aged hypertension with Epigallocatechin-3-gallate (EGCG) treatment (SHR-EGCG) group. **(A)** Representative Western blotting images of cytosolic AIF, mitochondrial AIF, cytosolic EndoG and mitochondrial EndoG in the cerebral cortex tissues from WKY, SHR, and SHR-EGCG groups with α-tubulin as an internal control. **(B)** Bars represent the protein quantification of cytosolic EndoG to mitochondrial EndoG as well as cytosolic AIF to mitochondrial AIF normalized by α-tubulin and COX IV, expressed as fold changes relative to the WKY group and mean values ± SEM (*n* = 6 in each group). **p < 0.01 and ***p < 0.001 in the comparison between the SHR group and WKY rat group; ##*p* < 0.01, in the comparison between the SHR-EGCG group and SHR group.

To evaluate the EGCG effects on neural EndoG and AIF-related Caspase-independent apoptotic pathway in early aged hypertension, Western blotting was carried out to evaluate the protein distributions of EndoG and AIF between mitochondria and cytosol in the cerebral cortex tissue among the WKY, SHR, and SHR-EGCG groups. The protein ratios of cytosolic EndoG to mitochondrial EndoG and cytosolic AIF to mitochondrial AIF in the SHR group were significantly greater than those in the WKY group. In contrast, those ratios in the SHR-EGCG group were significantly lower than those in the SHR group (Figure 3).

### Upstream Components of Fas-Mediated Caspase-Dependent Apoptotic Pathway

**FIGURE 4:**
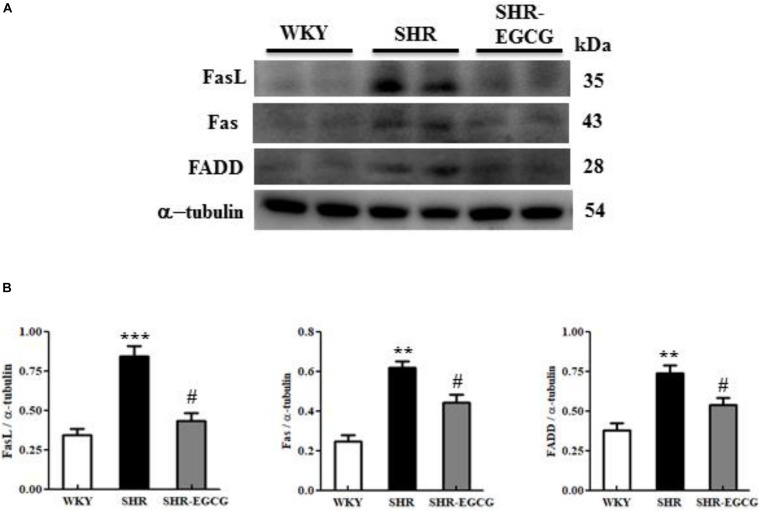
The upstream components of the Fas-mediated Caspase-dependent apoptotic pathway in a control Wistar Kyoto (WKY) group, a spontaneously early aged hypertensive (SHR) group and an early aged hypertension with Epigallocatechin-3-gallate (EGCG) treatment (SHR-EGCG) group. **(A)** Representative Western blotting images of Fas ligand (FasL), Fas receptor (Fas), and Fas-associated death domain (FADD) in the cerebral cortex tissues from the WKY, SHR, and SHR-EGCG groups with α-tubulin as an internal control. **(B)** Bars represent the protein quantification of FasL, Fas, and FADD normalized by α-tubulin, expressed as fold changes relative to the WKY group and mean values ± SEM (*n* = 6 in each group). **p < 0.01 and ***p < 0.001 in the comparison between the SHR group and the WKY rat group. #*p* < 0.05 and ##*p* < 0.01, in the comparison between SHR-EGCG group and SHR group.

In order to determine the EGCG effects on the upstream components of neural Fas receptor Caspase-dependent apoptotic pathway in early aged hypertension, we evaluated the protein expressions of FasL, Fas, and FADD in the cerebral cortex tissues from the WKY, SHR, and SHR-EGCG groups. The protein expressions of FasL, Fas, and FADD in the SHR group were significantly increased in comparison with the WKY group. However, those levels in the SHR-EGCG group were significantly reduced in comparison with the SHR group (Figure 4).

### Upstream Components of Mitochondrial-Mediated Caspase-Dependent Apoptotic Pathway and Bcl2 Family-Related Pro-survival Pathway

**FIGURE 5:**
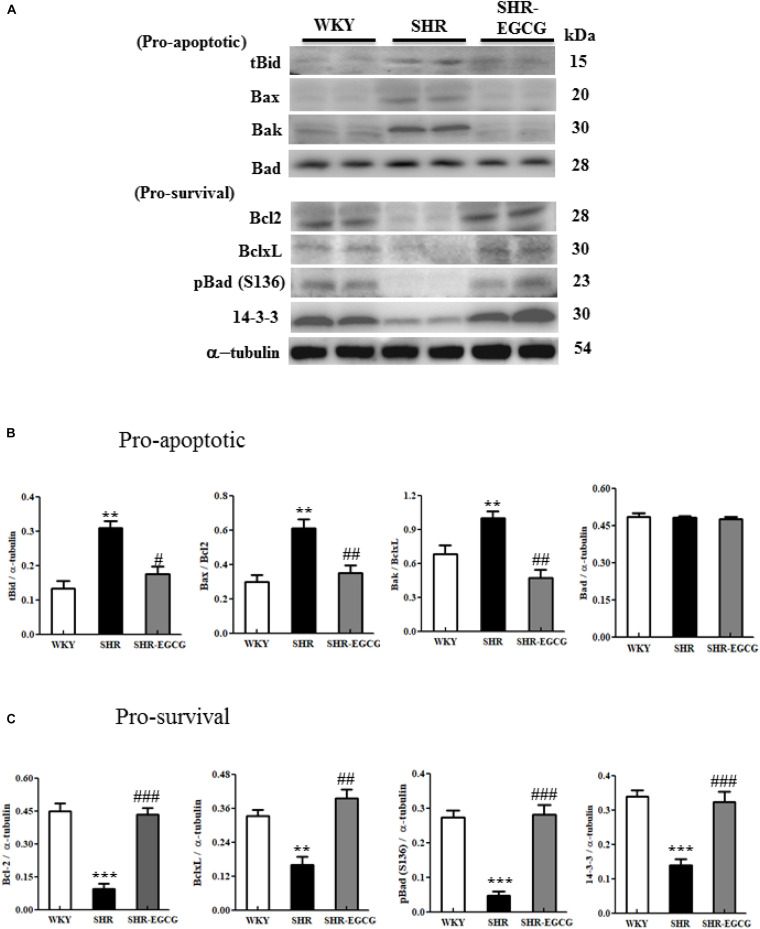
The upstream components of the mitochondrial-mediated Caspase-dependent apoptotic pathway in a control Wistar Kyoto (WKY) group, a spontaneously early aged hypertensive (SHR) group and an early aged hypertension with Epigallocatechin-3-gallate (EGCG) treatment (SHR-EGCG) group. **(A)** Representative Western blotting images of the truncated BH3 Interacting Domain Death Agonist (t-Bid), Bcl-2 associated protein X (Bax), Bcl-2 homologous antagonist/killer (Bak), Bcl-2 associated agonist of cell death (Bad), B-cell lymphoma 2 (Bcl-2), B-cell lymphoma-extra large (Bcl-xL), p-Bad, and 14-3-3 in the cerebral cortex tissues from the WKY, SHR, and SHR-EGCG groups with α-tubulin as an internal control. **(B,C)** Bars represent the protein quantification of t-Bid, Bax/Bcl-2, Bak/Bcl-xL, and Bad, as well as Bcl-2, Bcl-xL, pBad and 14-3-3 normalized by α-tubulin, expressed as fold changes relative to the WKY group and mean values ± SEM (*n* = 6 in each group). **p < 0.01 and ***p < 0.001, in comparison between the SHR and WKY rat group; #*p* < 0.05, ##*p* < 0.01, and ###*p* < 0.001 in comparison between the SHR-EGCG group and SHR group.

To determine the effects of EGCG on the upstream components of mitochondrial-mediated Caspase-dependent apoptotic pathway and Bcl-2 family-related pro-survival pathway in the early aged hypertensive brain, Bcl-2 family-related pro-apoptotic factors (t-Bid, Bax, Bak, and Bad) as well as Bcl-2 family-related pro-survival factors (Bcl-2, Bcl-xL, p-Bad and 14-3-3) expressions in cerebral cortex tissue from WKY, SHR, SHR-EGCG groups were evaluated by Western blotting. The levels of t-Bid, Bax/Bcl-2, Bak/Bcl-xL, but not Bad in the SHR group were significantly higher than those in the WKY group, whereas those levels in SHR-EGCG group were significantly lower than those in the SHR group. Bad levels were unchanged among the WKY, SHR, and SHR-EGCG groups. In contrast, the protein levels of Bcl-2, Bcl-xL, p-Bad, and 14-3-3, as well as pro-survival indices Bcl-2/Bax, Bcl-xL/Bak, and p-Bad/Bad, were significantly reduced in the SHR group when compared to the WKY group, whereas those indices were significantly increased in the SHR-EGCG group when compared to the SHR group (Figure 5).

### Downstream Components of Fas- Mediated and Mitochondrial-Mediated Caspase-Dependent Apoptotic Pathway

**FIGURE 6:**
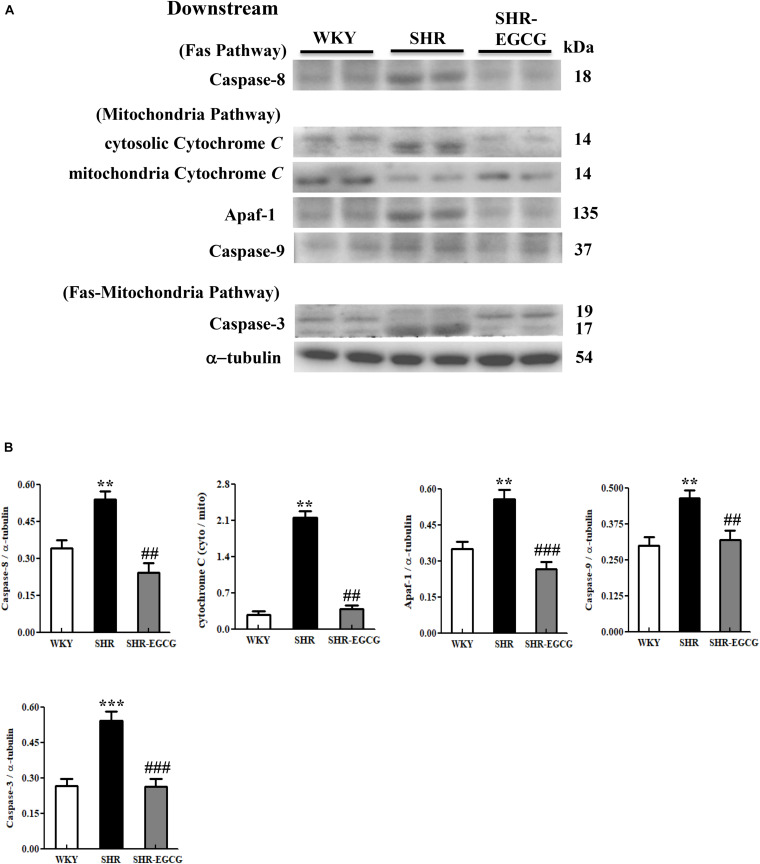
The downstream components of the Caspase dependent-apoptotic pathway in a control Wistar Kyoto (WKY) group, a spontaneously early aged hypertensive (SHR) group and an early aged hypertension with Epigallocatechin-3-gallate (EGCG) treatment (SHR-EGCG) group. **(A)** Representative Western blotting images of Caspase-8 (Fas downstream), cytosolic Cytochrome C, mitochondrial Cytochrome C, Apaf-1, Caspase-9 (Mitochondrial downstream), Caspase-3 (Fas and Mitochondrial downstream) in the cerebral cortex tissues from the WKY, SHR, and SHR-EGCG groups with α-tubulin as an internal control. **(B)** Bars represent the protein quantification of Caspase-8, cytosolic Cytochrome C to mitochondrial Cytochrome C ratio, Apaf-1, Caspase-9, Caspase-3 normalized by α-tubulin, expressed as fold changes relative to the WKY group and mean values ± SEM (*n* = 6 in each group). **p < 0.01 and ***p < 0.001 in comparison between the SHR group and WKY rat group; ##*p* < 0.01, ###*p* < 0.001 in comparison between the SHR-EGCG group and SHR group.

To determine whether EGCG attenuates downstream components of neural Fas-mediated and mitochondrial-mediated Caspase-dependent apoptotic pathways in early aged hypertension, Western blotting was carried out to compare the protein expressions of Caspase-8 (Fas-mediated), mitochondrial Cytochrome *C*, cytosolic Cytochrome C, Apaf-1, Caspase-9 (mitochondrial-mediated), and Caspase-3 (Fas-mediated and mitochondrial-mediated) in cerebral cortex tissues among WKY, SHR, and SHR-EGCG groups. The levels of Caspase-8 (Fas-mediated), Apaf-1, Caspase-9, cytosolic Cytochrome C to mitochondrial Cytochrome C ratio (mitochondrial-mediated), Caspase-3 (Fas-mediated and mitochondrial-mediated), in the SHR group were significantly increased when compared to the WKY group. However, those levels in the SHR-EGCG group were significantly reduced in comparison with the SHR group (Figure 6).

### Sirt1/PI3K/AKT-Related Pro-survival Pathway

**FIGURE 7:**
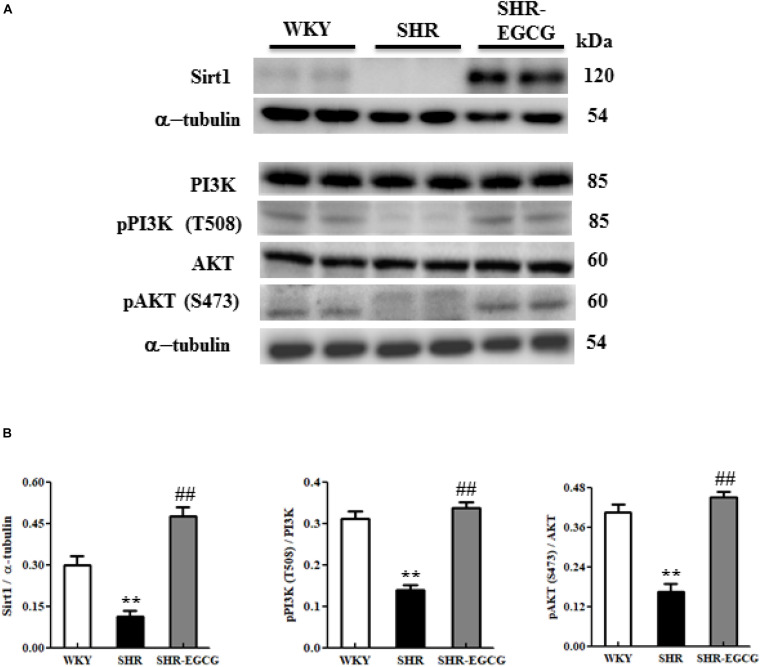
The Sirt1/PI3K/AKT-related survival pathway in a control Wistar Kyoto (WKY) group, a spontaneously early aged hypertensive (SHR) group, and an early aged hypertension with EGCG treatment (SHR-EGCG) group. **(A)** Representative Western blotting images of Sirt1, PI3K, p-PI3K, AKT, and p-AKT in the cerebral cortex tissues from the WKY, SHR, and SHR-EGCG groups with α-tubulin as an internal control. **(B)** Bars represent the protein quantification of Sirt1, p-PI3K/PI3K, and pAKT/AKT normalized by α-tubulin, expressed as fold changes relative to the WKY group and mean values ± SEM (*n* = 6 in each group). **p < 0.01, in comparison between the SHR group and WKY rat group; ##*p* < 0.01, in the comparison between the SHR-EGCG group and SHR group.

To evaluate the effects of EGCG on neural pro-survival pathway under early aged hypertension, the levels of Sirt1, PI3K, p-PI3K, AKT, and p-AKT in the cerebral cortex tissue from WKY, SHR, and SHR-EGCG groups were determined by Western blotting. The levels of Sirt1, p-PI3K to PI3K ratio and the p-AKT to AKT ratio in the SHR group were significantly reduced in comparison with the WKY group. However, those levels in the SHR-EGCG group were significantly increased when compared to the SHR group (Figure 7).

## Discussion

**FIGURE 8:**
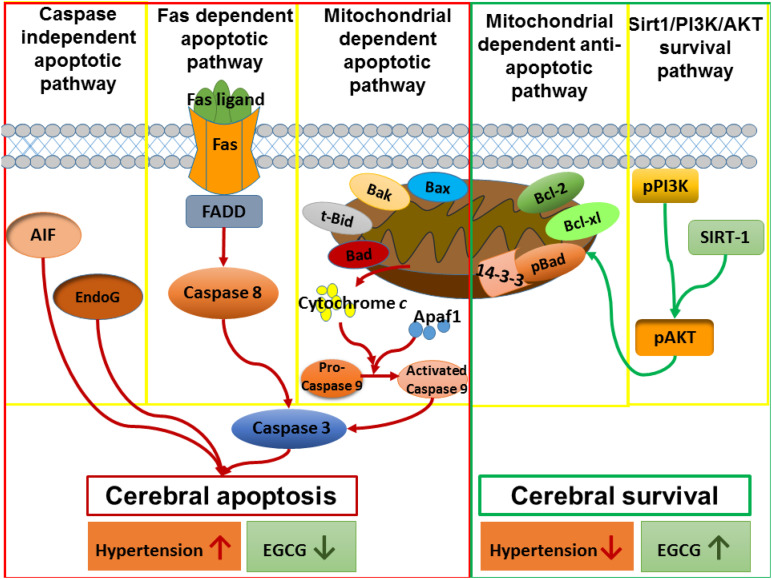
The hypothesized diagram. We proposed that the cerebral cortex EndoG and AIF-related Caspase-independent, Fas-mediated Caspase-dependent and mitochondrial-mediated Caspase-dependent apoptotic pathways were promoted in early aged hypertension, whereas those pathways were suppressed after EGCG treatment. This hypothesis was evidenced by which early aged hypertension increased the levels of EndoG, and AIF-related Caspase-independent apoptotic proteins (EndoG, AIF), Fas-mediated Caspase-dependent apoptotic proteins (FasL, Fas, FADD, Caspase-8, Caspase-3), and mitochondrial-mediated Caspase-dependent apoptotic proteins (Bax, Bak, t-Bid, Cytochrome C, Apaf-1, Caspase-9, Caspase-3), whereas EGCG treatment reduced those levels in the early aged hypertensive brain. On the contrary, the Bcl-2 family-related and Sirt1/PI3K/AKT related pro-survival pathways were inhibited in the early aged hypertensive brain, while those pathways were enhanced by EGCG treatment. This hypothesis was evidenced in which early aged hypertension suppressed Bcl-2 family-related pro-survival proteins (Bcl-2, Bcl-xL, pBad, 14-3-3) and Sirt1/PI3K/AKT related pro-survival proteins (Sirt1, p-PI3K/PI3K, p-AKT/AKT), whereas EGCG treatment enhanced those levels in the early aged hypertensive brain.

In the current study, the new findings were found as follows: (1) Early aged hypertension activated neural EndoG and AIF-related Caspase-independent, Fas-mediated Caspase-dependent, and mitochondrial-mediated Caspase-dependent apoptotic pathways as well as inhibited Bcl-2 family-related and Sirt1/PI3K/AKT related pro-survival pathways in the cerebral cortex. (2) EGCG treatment for 12 weeks decreased systolic blood pressure, diastolic blood pressure, and mean blood pressure in early aged hypertensive rats. (3) EGCG treatment reduced early aged hypertension-induced TUNEL positive cells in the cerebral cortex; (4) EGCG treatment reduced the early aged hypertension-activated EndoG and AIF-related Caspase-independent apoptotic pathway, in which the evidence was based on decreases in ratios of cytosolic EndoG to mitochondrial EndoG as well as cytosolic AIF to mitochondrial AIF. (5) EGCG treatment reduced early aged hypertension-activated Fas-mediated Caspase-dependent apoptotic pathway, in which the evidence was based on decreases in expression levels of FasL, Fas, FADD, Caspase-8, and Caspase-3 in the cerebral cortex. (6) EGCG treatment attenuated early aged hypertension-activated neural mitochondrial-mediated Caspase-dependent apoptotic pathway, in which the evidence was based on decreases in expression levels of t-Bid, Bax/Bcl-2, Bak/Bcl-xL, Apaf-1, cytosolic Cytochrome *C*, Caspase-9, and Caspase-3 in the cerebral cortex; (7) EGCG treatment enhanced Bcl-2 family-related pro-survival protein levels (Bcl-2, Bcl-xL, p-Bad, 14-3-3) and Sirt1/PI3K/AKT related pro-survival protein levels (Sirt1, p-PI3K/PI3K, p-AKT/AKT) in the early aged hypertensive cerebral cortex. Taken our findings and the previously apoptotic theories together, we drew the hypothesized diagram (Figure 8), which suggests that the cerebral cortex EndoG and AIF-related Caspase-independent, Fas-mediated Caspase-dependent and mitochondrial-mediated Caspase-dependent apoptotic pathways were augmented by early aged hypertension and were attenuated by EGCG treatment. In contrast, the cerebral cortex Bcl-2 family-related and Sirt1/PI3K/AKT related pro-survival pathways were suppressed by early aged hypertension and were enhanced after EGCG treatment. Our study found that EGCG appeared to attenuate neural apoptosis and enhance neural survival in the early aged hypertensive cerebral cortex.

Chronic hypertension or aged hypertension is a high-risk factor of neurodegeneration (Kruyer et al., 2015). Neural apoptosis of the brain was augmented by oxidative stress in hypertension or aged hypertension (Poulet et al., 2006; Li et al., 2012). A previous study showed that the Bax/Bcl-2 ratio was increased in the mature hypertensive cerebral cortex (Li et al., 2012). Another study observed the elevated levels of Bax and Cytochrome *C* proteins along with the reduced levels of Bcl-2 protein in the mature or aged hypertensive brain when compared to the age-match normal brain (Li et al., 2016). Besides, hypertension has been shown to reduce the protein levels of PI3K and p-AKT in the cerebral cortex of stroke-prone spontaneously hypertensive rats (Yoshitomi et al., 2011). Consistent with those data, we found that early aged hypertension not only activated the mitochondrial-mediated Caspase-dependent apoptotic pathway but also inhibited Bcl-2 family-related and PI3K/AKT related pro-survival pathways in the cerebral cortex. Furthermore, our study additionally clarified that early aged hypertension also augmented the EndoG and AIF-related Caspase-independent and Fas-mediated Caspase-dependent apoptotic pathways. This new finding added more evidence of hypertension-induced neural apoptosis *via* EndoG/AIF-related and Fas-mediated pathways.

Our study found that the systolic blood pressure, diastolic blood pressure, and mean blood pressure in hypertensive rats were reduced by 12 weeks of EGCG treatment. In agreement with our findings, a previous study showed that EGCG treatment decreased blood pressure *via* balancing neurotransmitters and cytokines in the hypothalamic paraventricular nucleus (Yi et al., 2016). Another study observed that EGCG treatment attenuated the endothelial dysfunction in vessels, leading to reduced blood pressure in spontaneously hypertensive rats with myocardial ischemia/reperfusion injury (Potenza et al., 2007). Besides, a study suggested that the renin-angiotensin system, a major hormone system that regulates blood pressure, was inhibited by EGCG treatment *in vitro* (Li et al., 2013). Taken together, our findings supported that EGCG treatment could reduce blood pressure.

The current study demonstrated that EGCG treatment reduced the percentage of neural cell deaths in the early aged hypertensive cerebral cortex, suggesting that EGCG treatment could attenuate neural deterioration in the cerebral cortex against early aged hypertension. Consistent with our study, a previous study reported that 20 mg/kg EGCG intraperitoneal administration suppressed the TUNEL-positive neural cells in the brain of ischemic stroke rats (Nan et al., 2018). Additionally, another study observed that 2 mg/kg/day EGCG subcutaneous injection attenuated the TUNEL-positive hippocampal cells in aging mice (He et al., 2009). Therefore, we confirmed that EGCG treatment could provide the protective effects on neural cells in the cerebral cortex under early aged hypertension.

As reported in this study, EGCG treatment prevented overactive EndoG and AIF-related Caspase-independent apoptotic pathway in early aged hypertension, as evidenced by reductions in the ratios of cytosolic EndoG to mitochondrial EndoG as well as cytosolic AIF to mitochondrial AIF proteins in the early aged hypertensive cerebral cortex after EGCG treatment, suggesting that EGCG treatment could alleviate the translocation of EndoG and AIF from neural mitochondria to neural cytosol. Our study is the first to report the EGCG treatment attenuates early aged hypertension-induced EndoG and AIF caspase-independent pathway in the cerebral cortex. A previous study investigating different organs with different disease models has reported that EGCG treatment reduced the level of AIF protein in the rats model with nicotine-induced cardiac apoptosis (Nacera et al., 2017). Of note, due to its permeability through the brain-blood barrier (Bastianetto et al., 2011), EGCG treatment could directly adjust neuronal mitochondrial membrane permeability and calcium homeostasis, which thereby may prevent the release of EndoG and AIF from mitochondria and inactivate Caspase-independent apoptotic pathway (Campos-Esparza et al., 2009).

Our results indicated the new finding that EGCG treatment was able to prevent the early aged hypertension-activated Fas-mediated Caspase-dependent neural apoptotic pathway, as evidenced by the down-regulated levels of FasL, Fas, FADD, Caspase-8, and Caspase-3 in the hypertensive cerebral cortex. Supportively, a previous study observed that 1 μM EGCG treatment reduced the mRNA level of Fas ligand in the neuronal cell line (SH-SY5Y cells) *in vitro* (Weinreb et al., 2003). In addition, EGCG has been reported to decrease Caspase-8 protein levels in radiation-induced hippocampal apoptosis *in vivo* (El-Missiry et al., 2018) and nitrite oxide-induced neuronal cell death (PC12 cells) *in vitro* (Jung et al., 2007). Therefore, EGCG treatment could diminish the early aged hypertension-activated Fas-mediated Caspase-dependent neural apoptotic pathway in the cerebral cortex.

In the present study, we found that EGCG inactivated both upstream components (t-Bid, Bax/Bcl-2, and Bak/Bcl-xL) and downstream components (cytosolic Cytochrome *C*, Apaf-1, Caspase-9, Caspase-3) of the mitochondrial-mediated Caspase-dependent apoptotic pathway in the early aged hypertensive cerebral cortex. Supportively, previous studies showed that EGCG attenuated the overexpression of Bax, Caspase-9, Cytochrome *C* protein levels in the radiation-induced hippocampal apoptosis *in vivo* (El-Missiry et al., 2018) and nitrite oxide-induced neuronal cell death (PC12 cells) *in vitro* (Jung et al., 2007). Another study observed that EGCG decreased the Bax protein level in the cerebral ischemia brain (Nan et al., 2018). Hence, we suggested that EGCG could significantly reduce the early aged hypertension-activated mitochondrial-mediated Caspase-dependent apoptotic pathway in the brain.

We identified that EGCG treatment activated the Bcl-2 family-related pro-survival pathway against early aged hypertension in the cerebral cortex, as evidenced by the up-regulated levels of Bcl-2, Bcl-xL, pBad, and 14-3-3. Although there was no previous evidence that mentioned the effects of EGCG on Bcl-2 family-related pro-survival pathway in the early aged hypertensive brain, previous studies showed that EGCG enhanced Bcl-2 protein levels in the cerebral ischemia brain (Nan et al., 2018) and in radiation-induced hippocampal apoptosis (El-Missiry et al., 2018). Likewise, EGCG has been reported to increase the mRNA and protein levels of Bcl-2 and Bcl-xL in quinolinic acid-induced neuronal cell death (N18D3 cells) *in vitro* (Jang et al., 2010). Those data suggested that EGCG treatment could enhance the Bcl-2 pro-survival pathway in the brain against early aged hypertension.

Moreover, in this study, EGCG activated the Sirt1/PI3K/AKT pro-survival pathway by increasing Sirt1 levels as well as promoting the phosphorylation of PI3K and AKT in the hypertensive cerebral cortex. Supportively, a study has shown that EGCG reduced neural apoptosis in the cerebral ischemic brain by enhancing the neural PI3K/AKT pathway (Nan et al., 2018). Another study reported that the activation of the PI3K/AKT pathway by EGCG protected the brain against amyloid-beta accumulation, which thereby slowed down Alzheimer’s disease progression (Yamamoto et al., 2017). In addition, EGCG has been shown to directly improve neural survival in the aging hippocampus, both *in vivo* and *in vitro*, by enhancing the PI3K/AKT pathway (Ortiz-López et al., 2016). Therefore, we hypothesized that EGCG not only reduced neural cell loss but also enhanced neural survival in the early aged hypertensive cerebral cortex through regulating the Sirt1/PI3K/AKT pathway. Of note, Sirt1 not only activates p-AKT, but also play a key role in mitochondrial quality-control. The activation of Sirt1 might attenuate the damage on neural mitochondrial biogenesis, and thus, mitigate neural apoptosis in the cerebral cortex (Li and Wang, 2017; Teertam and Prakash Babu, 2021). This question need to be addressed in further studies.

### Study’s Limitation

There were several limitations in our study which need to be mentioned. Firstly, 6% of unknown micronutrients in the chosen-EGCG product (TEAVAGO) might induce the apoptotic effects on the hypertensive cerebral cortex, leading to reduce the anti-apoptotic effects of EGCG treatment observed in this study. Secondly, the TUNEL assay cannot distinguish apoptosis from necrosis, and thus, we were unable to rule out hypertension-related neural necrotic cell deaths. Finally, the effects of EGCG on neural apoptosis in the early aged hypertensive cerebral cortex noted here cannot be distinguished or attributed to specific factors because EGCG treatment affects multiple factors such as anti-hypertension, enhanced-neuronal stem cells, anti-inflammation, anti-oxidative stress, and baroreflex regulation (Itoh et al., 2013; Legeay et al., 2015; Li et al., 2019). Our study proved the therapeutic effects of EGCG on early aged hypertension-induced neural apoptosis in the cerebral cortex, but cannot provide the cause-effect of why EGCG treatment could reduce neural apoptosis and enhance neural survival in the early aged hypertensive cerebral cortex. Thus, the mechanisms of EGCG against neural apoptosis in the early aged hypertensive brain need to be clarified in further studies.

### Conclusion and Perspective

Since early aged hypertension augments neural apoptosis in the cerebral cortex, chronic hypertensive patients should be aware of the progressive development of neurodegenerative diseases. It is difficult to extract the cerebral cortex tissues from the human brain, and thus, the cerebral cortex in the spontaneously early aged hypertensive rats with EGCG treatment here should provide a critical explanation on how EGCG treatment prevents apoptosis-related cerebral cortex disorders in hypertensive patients. If EGCG prevents the neural apoptosis in the hypertensive cerebral cortex, EGCG may slow down the progression of early aged hypertension-induced neurodegeneration. Our findings demonstrated that EGCG reduced hypertensive-augmented neural apoptosis in the cerebral cortex through preventing overactive neural EndoG and AIF-related Caspase-independent apoptotic pathways, Fas-mediated Caspase-dependent apoptotic pathway and mitochondrial-mediated Caspase-dependent apoptotic pathways as well as enhancing the cerebral cortex Bcl2-related and PI3K/AKT-related pro-survival pathways. Thus, we might further hypothesize that EGCG represents the natural therapeutic agent for attenuating early aged hypertension-induced cerebral cortex apoptosis, which might prevent the early aged hypertension-augmented neurodegeneration. Regarding clinical applications, although EGCG treatment reportedly reduced blood pressure and improved neurobehavioral performance in patients (Legeay et al., 2015; Cascella et al., 2017), the dosage of EGCG used was varied. Additionally, it was unclear whether EGCG treatment could reduce neurodegenerative progression in early aged hypertensive patients. Therefore, clinical studies are required to determine the long-term effects of EGCG treatment with dosage comparison in neurodegenerative patients.

## Data Availability Statement

The datasets presented in this study can be found in online repositories. The names of the repository/repositories and accession number(s) can be found below: https://1drv.ms/x/s!Ag29XwtG6G8vg8sqI-Zhf9FiX1DtgQ.

## Ethics Statement

The animal study was reviewed and approved by Institutional Animal Care and Use Committee of China Medical University.

## Author Contributions

S-DL, Z-YC, A-LY, and X-BW: conceptualization. M-HH, S-YT, A-LY, and S-DL: experimental resources. M-HH, S-YT, Y-YL, and A-LY: performing the experiments. M-HH, S-YT, and S-DL: data analysis. NTN, S-HY, and Y-JC: writing—original draft preparation. S-DL, NTN, and Z-YC: writing—review and editing. All authors have read and agreed to the published version of the manuscript.

## Conflict of Interest

The authors declare that the research was conducted in the absence of any commercial or financial relationships that could be construed as a potential conflict of interest.

## Publisher’s Note

All claims expressed in this article are solely those of the authors and do not necessarily represent those of their affiliated organizations, or those of the publisher, the editors and the reviewers. Any product that may be evaluated in this article, or claim that may be made by its manufacturer, is not guaranteed or endorsed by the publisher.
